# The Effect of m6A Methylation Regulatory Factors on the Malignant Progression and Clinical Prognosis of Hepatocellular Carcinoma

**DOI:** 10.3389/fonc.2020.01435

**Published:** 2020-08-21

**Authors:** Zhongwei Zhao, Lili Yang, Shiji Fang, Liyun Zheng, Fazong Wu, Weiqian Chen, Jingjing Song, Minjiang Chen, Jiansong Ji

**Affiliations:** ^1^Zhejiang Provincial Key Laboratory of Imaging Diagnosis and Minimally Invasive Intervention Research, The Fifth Affiliated Hospital of Wenzhou Medical University, Lishui Hospital of Zhejiang University, Lishui Municipal Central Hospital, Lishui, China; ^2^Department of Anesthesiology, The Fifth Affiliated Hospital of Wenzhou Medical University, Lishui Hospital of Zhejiang University, Lishui Municipal Central Hospital, Lishui, China

**Keywords:** N^6^-methyladenosine (m6A), malignant progression of HCC, prognosis, Cox regression, consensus clustering

## Abstract

The modification level of the transcript N^6^-methyladenosine (m6A), dynamically regulated by methyltransferases, binding proteins and demethylases, is closely related to the occurrence, and progression of tumors. Here, 13 differentially expressed m6A methylation regulators were confirmed in 374 hepatocellular carcinoma (HCC) patients, among which RBM15, YTHDC1, YTHDF1, and YTHDF2 were significantly variant in different stages and grades. Further consensus clustering analysis identified two HCC subtypes (cluster1/2) in this cohort, finding an active role of the m6A methylation regulators in the malignant progression of HCC. Furthermore, GESA enrichment analysis showed that PPAR signaling pathway, and the pathways involved in retinol metabolism and peroxisome were related to tumor progression. Additionally, a 4-gene risk model (ROC = 0.729) that can be used as a prognostic marker and a predictor for clinicopathological characteristics of HCC was constructed via univariate and multivariate Cox regression analyses. Analysis on overall survival and disease-free survival demonstrated that METTL3 and YTHDF1 out of the four genes in the model could serve as independent prognostic factors for HCC. Overall, this study systematically investigated the effect of m6A methylation regulators on the malignant progression of HCC and proposed a 4-gene risk prediction model, laying a theoretical foundation for the further research on HCC prognosis.

## Highlights

- The expression, potential function, and prognostic value of m6A methylation regulatory factors in hepatocellular carcinoma were systematically studied for the first time;
- The relationship between m6A methylation regulatory factors and clinical features of hepatocellular carcinoma was systematically studied for the first time.

## Introduction

More than 160 kinds of RNA modification have been found in organisms as a kind of post-transcriptional regulation (1). N^6^-methyladenosine (m6A) is the most common type of mRNA modification accounting for about 50% of the total methylated nucleotides, and 0.1–0.4% of adenine in cells can be methylated, with an average of 3–5 methylation sites on every strand of mRNA (2–5). The m6A sites are enriched near the termination codon and in the 3′UTR, and there is a correlation between m6A residues and miRNA binding sites in the 3′UTR (6). m6A RNA (7) affects RNA splicing, translation and stability through Capable-dependent translation in 5′UTR.

The modification level of the transcript m6A is dynamically regulated by “writers” (methyltransferases), “readers” (binding proteins), and “erasers” (demethylases). The core components of m6A methyltransferase complex include METTL3, METTL14, and WTAP, among which METTL3 acts as a core catalyst, METTL14 functions as a structural support for METTL3, and WTAP stabilizes core complex (8–12). In addition, VIRMA, RBM15, and ZC3H13 act as regulatory subunits to modulate the intracellular activity of METTL3 (13, 14). The currently identified m6A RNA “erasers” include FTO and ALKBH5, both of which belong to the ALKB dioxygenase family proteins and have catalytic function depending on cofactor Fe^2+^/α-Ketoglutaric acid. FTO is the earliest discovered m6A RNA demethylase, which first reveals the reversibility of RNA chemical modification (15). ALKBH5 co-localizes with nuclear speckles in a RNase A-sensitive manner, which can directly catalyze m6A methylated adenosine to remove methylation without producing intermediate products (16). “Readers” can decode m6A methylation and generate functional signals, including the YTH family members (YTHDC1, YTHDF2, YTHDF1, THDF3, YTHDC2, and Mrb1, etc.), ELAVL1, FMR1, LRPPRC, and IGF2BP, etc. (17–20).

Recent studies find that RNA modification dynamically and reversibly regulates important biological functions such as RNA metabolism, processing, directional differentiation of stem cells and tumor development (21–24). Research discovered that METTL3 may be related to the occurrence and development of acute myeloid leukemia (AML) since that METTL3 has different effects on the proliferation and growth of various AML cell lines in mice and humans, and its down-regulation can promote the differentiation of AML cells (25, 26). It has been reported that m6A RNA methylation is significantly reduced in cervical squamous cell carcinoma, and its methylation level is associated with poor prognosis (27, 28). High m6A RNA methylation level promotes the development of liver cancer through increasing the expression of METTL3 and YTHDF2 while inhibiting SOCS2 expression (29). In this study, FPKM data (*n* = 424) and clinical information were obtained from The Cancer Genome Atlas (TCGA) database, and the role of the 13 widely applied m6A methylation-related factors in the malignant progression of hepatocellular carcinoma (HCC) was analyzed systematically. A 4-gene risk model was established and used to conduct stratification analysis on the prognosis of HCC and search for independent prognostic factors for HCC.

## Materials and Methods

### Dataset

FPKM data and clinicopathologic information of HCC were downloaded from TCGA database (http://cancergenome.nih.gov/), including 50 normal samples and 374 HCC samples.

### Correlation Between the Expression of m6A Methylation Regulatory Factors and Clinical Features

Based on the FPKM data and clinical information accessed from the TCGA-LIHC dataset, expression files of the 13 widely reported m6A methylation regulatory factors (METTL3, METTL14, FTO, ZC3H13, YTHDC1, YTHDC2, ALKBH5, WTAP, KIAA1429, RBM15, YTHDF1, YTHDF2, HNRNPC) were obtained. Wilcox test was used to analyze the differential expression of the m6A methylation regulatory factors in tumor group and normal group as well as in different clinicopathological periods.

### Consensus Clustering Analysis

The consensus clustering analysis of the samples was conducted by “ConsensusClusterPlus” package based on the expression levels of the m6A methylation regulatory factors. K was gradually increased from 2 to 9 to determine the optimal clustering model, and the subtypes of HCC were determined by clustering analysis. Based on the subtypes, Kaplan-Meier overall survival analysis, differential analysis of the expression of the methylation regulatory factors as well as patient's pathological stages were performed to detect the relationship between m6A methylation regulatory factors and the progression of HCC. In addition, GSEA pathway enrichment analysis of m6A methylation regulatory factors was conducted to determine the possible signaling pathways involved in the regulation of m6A methylation regulatory factors on HCC progression.

### Cox Regression Analysis

Univariate and multivariate Cox regression analyses were used to construct a risk model, and receiver operating characteristic (ROC) curve was used to test the model accuracy and screen prognosis-associated genes. Each patient was given a risk score based on the risk model and the samples were divided into high-risk and low-risk groups according to the median value of the risk score. Kaplan-Meier analysis was conducted to test the difference in the overall survival (OS) rate of the high and low-risk groups to further verify the effectiveness of the model. Effect of the m6A methylation regulatory factors in the risk model on the survival of patients was searched in the GEPIA database (including 370 clinical samples), and Kaplan-Meier OS and disease-free survival (DFS) curves were plotted. ROC analysis was further performed to determine whether the m6A methylation regulatory factors in the risk model could be used as independent prognostic factors for HCC. Clinical samples in the LIRI-JP dataset from the ICGC database were used as the validation set (including 232 clinical samples) to verify the accuracy of the risk model.

## Results

### Relationship Between the Expression of m6A RNA Methylation Regulatory Factors and Clinicopathological Features in HCC

**Figure 1:**
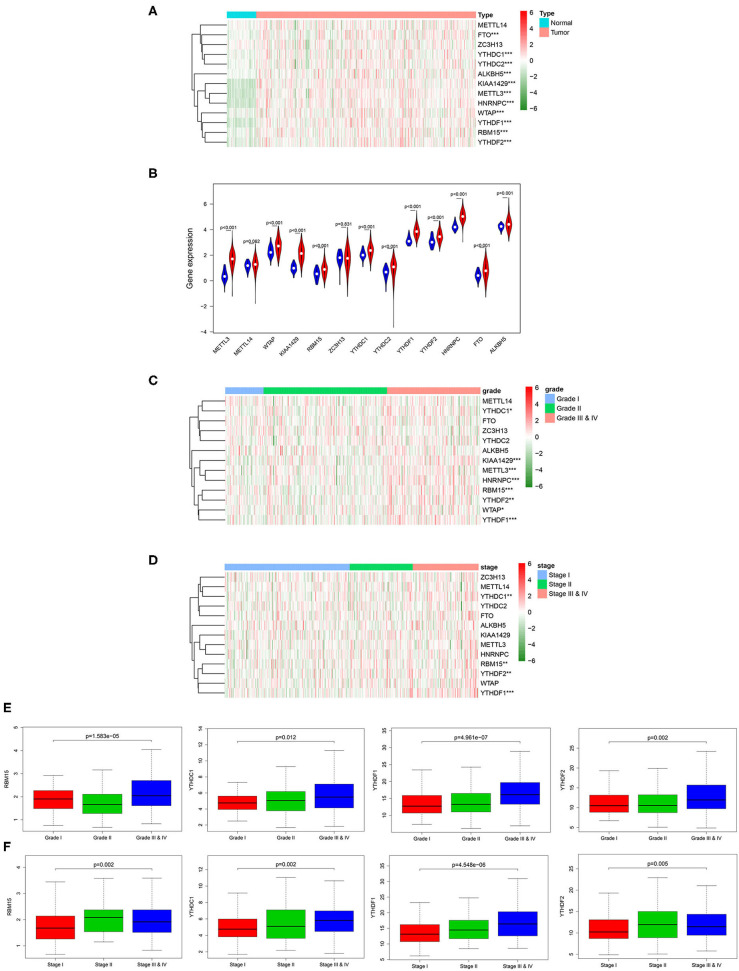
Relationship between the expression of m6A RNA methylation regulatory factors and clinicopathological features in HCC. **(A)** Heat map shows the expression of the 13 m6A methylation regulatory factors in tumor group and normal group. **(B)** The violin plot exhibits the expression of the 13 m6A methylation regulatory factors in tumor group (red) and normal group (blue). **(C,D)** Heat maps of 13 m6A methylation regulatory factors in different grades and stages. **(E,F)** Boxplots display the expression of RBM15, YTHDC1, YTHDF1 and YTHDF2 in different grades and stages. ***p < 0.001, **p < 0.01, *p < 0.05.

m6A RNA methylation modification is a dynamic regulatory process in the occurrence and development of tumor, and every m6A RNA methylation regulatory factor plays an important biological role. Therefore, we analyzed the relationship between the expression of the 13 widely reported methylation regulatory factors (METTL3, METTL14, FTO, ZC3H13, YTHDC1, YTHDC2, ALKBH5, WTAP, KIAA1429, RBM15, YTHDF1, YTHDF2, HNRNPC) and the clinicopathologic features systematically. Firstly, the differential expression of the m6A methylation regulatory factors in tumor group and normal group was analyzed through Wilcox test, and it was exhibited that almost all m6A methylation regulatory factors expressed differentially in tumor group and normal group (Figures 1A,B). After that, we further analyzed the correlation between the expression of methylation regulatory factors and the clinicopathological features, and discovered that four genes (RBM15, YTHDC1, YTHDF1, and YTHDF2) showed significant differential expression in different stages and grades, and their expression levels gradually increased with the progression of the disease (Figures 1C–F).

### Relationship Between m6A Methylation Regulatory Factors and Malignant Progression of Tumor

**Figure 2:**
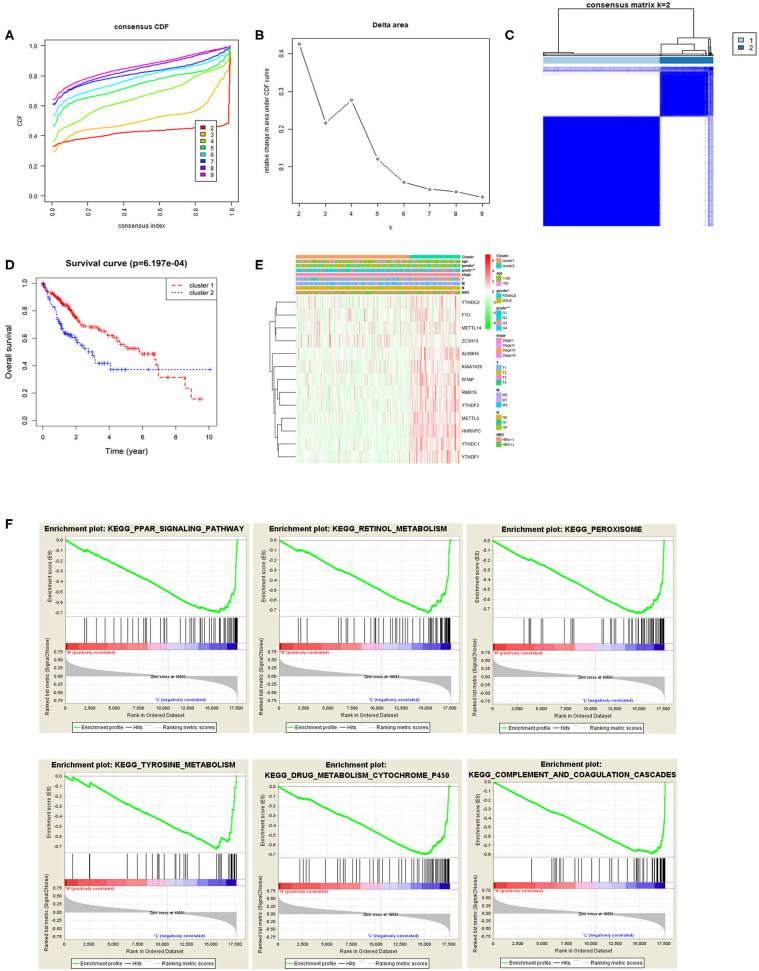
Relationship between m6A methylation regulatory factors and the malignant progression of tumor. **(A,B)** Cumulative distribution function (CDF) of consensus clustering analysis, and the relative change in the area under the CDF curves when k = 2–9. **(C)** The samples were divided into cluster1 and cluster2 when k = 2, with a high intra-group correlation and a low or no significant intergroup correlation. **(D)** The Kaplan-Meier curves show the OS of patients in the two groups. **(E)** Heat map displays the difference in the expression of m6A methylation regulatory factors and the difference in clinicopathological features between the two groups. **(F)** KEGG enrichment analysis on the m6A methylation regulatory factors in the two groups.

Based on the expression similarity of m6A methylation regulatory factors, consensus clustering analysis was conducted on the samples. During the process of K increasing from 2 to 9, the optimal clustering model was found when K = 2 with a high intra-group correlation and a low or no significant intergroup correlation (Figures 2A–C). Two cancer subtypes (cluster1 and cluster2) were identified and Kaplan-Meier analysis suggested that the survival time of the patients in the cluster2 group was significantly shorter than that of the patients in the cluster1 group (Figure 2D). Heat map exhibited that the expression levels of the m6A methylation regulatory factors in the cluster2 group were significantly higher than those in the cluster1 group, and there were significant differences between the two groups in pathological grade and stage (Figure 2E). Moreover, it further proved that m6A methylation regulatory factors could promote the malignant progression of HCC. KEGG pathway enrichment analysis was performed on the genes in two groups by GSEA software, and it was noted that the regulatory factors were mainly enriched in the PPAR signaling pathway, and the pathways related to retinol metabolism, peroxisome, tyrosine metabolism, drug metabolism cytochrome P450, complement and blood coagulation cascade and so on (Figure 2F), suggesting that there was a close relationship between these regulatory factors and signaling pathways.

### m6A Methylation Regulatory Factors Can Be Used as Independent Prognostic Factors for HCC Patients

**Figure 3:**
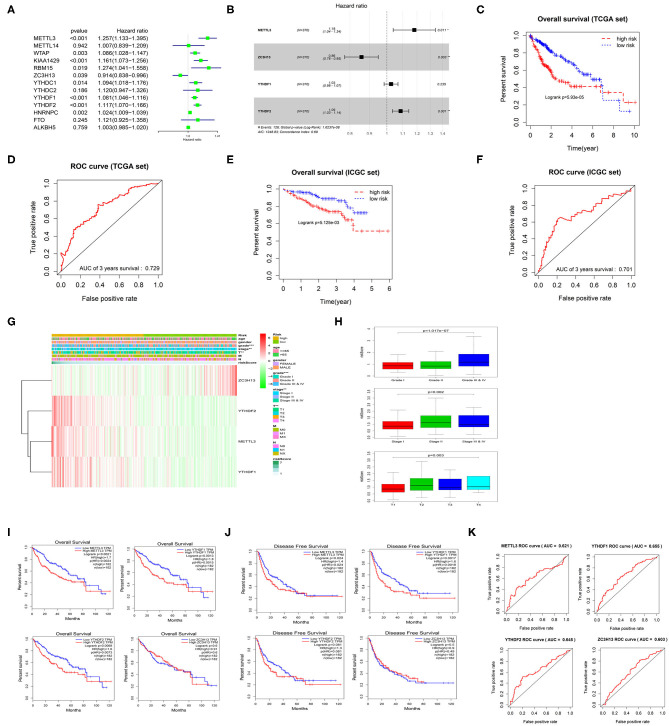
m6A methylation regulatory factors can be used as independent prognostic factors for HCC patients. **(A)** Univariate Cox regression analysis of m6A methylation regulatory factors. **(B)** Multivariate Cox regression analysis of the prognosis-related feature genes. **(C)** Kaplan-Meier OS analysis of the patients in high- and low-risk groups (median risk score as the threshold) in TCGA dataset. **(D)** ROC curves based on the risk model. **(E)** Kaplan-Meier OS curves for the patients in high- and low-risk groups in the LIRI-JP dataset from ICGC database. **(F)** ROC curves based on the risk model in the validation set. **(G)** The difference in the expression of the m6A methylation regulatory factors in the risk model and the difference in clinicopathological differences between the high- and low-risk groups are exhibited in an heatmap. **(H)** The relationship between the risk score and different clinical grades, stages and T stages of HCC. **(I,J)** Kaplan-Meier OS curves and DFS curves of the m6A methylation regulatory factors in the risk model are retrieved from GEPIA database. **(K)** ROC curves shows the potential of the 4 m6A methylation regulatory factors in the risk model as independent factors for HCC prognosis.

Univariate Cox regression analysis was performed on the 13 m6A methylation regulatory factors, and 9 genes significantly correlated with HCC prognosis were screened (Figure 3A). Thereafter, a 4-gene risk model consisting of ZC3H13, METTL3, YTHDF1, and YTHDF2 was constructed by multivariate Cox regression analysis on the nine feature genes (Figure 3B). ZC3H13 was a low-risk factor (HR < 1), while METTL3, YTHDF1, and YTHDF2 were high-risk factors (HR > 1). The samples were divided into the high-risk group and the low-risk group based on the median risk score. The results of survival analysis exhibited that the survival time of the patients in the high-risk group was significantly shorter than that in the low-risk group (Figure 3C). ROC analysis showed that the area under the curve (AUC) value of the model was 0.729 (Figure 3D), indicating a high accuracy of the model. Heat map suggested that with the increase in the risk score, the expression of ZC3H13 was gradually decreased while that of METTL3, YTHDF1, and YTHDF2 was increased. To further verify the accuracy of the risk model, we also used the prognostic data of the clinical samples in ICGC database. The results showed that the risk assessment model could still distinguish the survival of high- and low-risk patients with high accuracy in the validation set (AUC = 0.701) (Figures 3E,F). In addition, there were significant differences between the high-risk group and the low-risk group in different pathological grades and stages (Figure 3G), and the risk score was increased with tumor progression (Figure 3H), which further confirmed the reliability of the risk model. Kaplan-Meier OS curves and DFS curves of the m6A methylation regulatory factors in the risk model were plotted through GEPIA database. It was found that the expression of METTL3, YTHDF1, YTHDF2 had a significant effect on the OS of patients (Figure 3I), and the expression of METTL3 and YTHDF1 was significantly associated with DFS (Figure 3J). ROC analysis was carried out on the four genes in the risk model, respectively, and it was revealed that the AUC value of each gene was above 0.6 (Figure 3K). Therefore, it could be concluded that METTL3 and YTHDF1 could serve as independent prognostic factors for HCC.

## Discussion

HCC is the sixth most common cancer in the world and the fourth most common cause of cancer-related deaths, but its molecular mechanism is not yet clear (30). Studies have reported that genetic variation and epigenetic modification can affect the progression of tumors (31). However, the current studies on epigenetic mechanisms mainly focus on DNA methylation and histone modification, which have been found to affect the occurrence, progression and prognosis of HCC (32–37). m6A methylation is a newly discovered method of RNA chemical modification, and its role in HCC will be gradually revealed.

Among m6A methylation regulatory factors, WTAP has been found to be dramatically up-regulated in HCC, and WTAP-guided m6A modification promotes HCC progression through the Hur-ETS1-p21/p27 axis (38). Up-regulated METTL3 promotes the malignant progression of HCC by inhibiting SOCS2 expression through a m6A-YTHDF2-dependent mechanism (29). At the same time, studies suggest that miR-145 regulates m6A level by targeting the 3′-UTR of YTHDF2 mRNA in HCC cells (39). These studies expands the exploration of HCC and provides new ideas and directions for the clinical treatment and molecular diagnosis of HCC. However, studies on the relationship between m6A methylation regulatory factors and HCC are scarce at present.

In this study, we found that the expression levels of m6A methylation regulatory factors were correlated with the malignancy and prognosis of HCC. Meanwhile, we determined two HCC subtypes (cluster1/2) by consensus clustering analysis according to the expression similarity of m6A methylation regulatory factors. Cluster1/2 subtypes were found to be not only significantly different in patient's prognosis and different pathological stages, but were also seen to be closely related to the key signaling pathways implicated with the malignant progression of HCC. Besides, we constructed a 4-gene risk model, divided the samples into high- and low-risk groups according to the median risk score, and concluded that m6A methylation regulatory factors could function as prognostic factors for HCC.

RBM15, YTHDC1, YTHDF1, and YTHDF2 were found to be differentially expressed in the different stages of HCC patients in this study. RBM15 as a “writer” has been found to be differentially expressed in the head and neck squamous cell carcinoma, gastric cancer and colorectal adenocarcinoma (40–42). RBM15 can also function as an independent prognostic marker and a predictor for clinical pathological features of gastric cancer. YTHDC1, YTHDF1, and YTHDF2 are regarded as “readers,” among which YTHDF1 can promote the ribosome loading of m6A-modified mRNA molecules and improve the translation efficiency of target mRNA by interacting with translation initiation factors (17). YTHDF2 can competitively bind to methylated transcripts with ribosomal RNA (rRNA) that mediates mRNA translation, thereby affecting the half-life of RNA, accelerating RNA degradation, and affecting mRNA stability (18). YTHDC1 regulates the splicing of exons containing m6A by interacting with mRNA splicing factors (43). It is reported that YTHDF1 and HNRNPC can be used as prognostic factors for colon cancer and have potential value for colon cancer treatment (41). Silencing YTHDF2 can promote the inflammation, angiogenesis and metastasis of HCC (44). Our study demonstrated that RBM15, YTHDC1, YTHDF1, and YTHDF2 were significantly differentially expressed at different pathologic stages and grades of HCC, while the expression levels of YTHDC1 and YTHDF1 were increased gradually with the progression of disease. Combined with the above results, the expression of m6A RNA methylation regulatory factors is seen to be closely related to the malignant clinicopathological features of HCC.

In addition, we further explored that m6A RNA methylation regulatory factors were also associated with the biological processes and signaling pathways involved in the malignant progression of HCC. Zhao et al. found that the expression of YTHDF1 is significantly upregulated in patients with TNM stage III and IV HCC compared with that in patients with stage II HCC, and they have worse prognosis (45). Moreover, the potential target genes regulated by YTHDF1 protein may be related to tumor cell cycle, degradation of various amino acids and lipid metabolism, while the abnormal physiological functions may be related to the occurrence and development of HCC (45). In this study, two tumor subtypes (cluster1/2) were obtained through consensus clustering analysis, and KEGG analysis found that m6A methylation regulatory factors were enriched in the PPAR signaling pathway, and the pathways involved in retinol metabolism, peroxisome, tyrosine metabolism, drug metabolism cytochrome P450, complement and blood coagulation cascade. In the study of liver cancer biomarkers, researchers also found that some biomarkers related to the diagnosis of HCC are often enriched in PPAR signaling pathway and the pathway associated with retinol metabolism. Li et al. found that PPAR signaling pathway and retinol metabolism are the two pathways where differentially expressed long non-coding RNAs (DElncRNAs) remarkably enriched, while these DElncRNAs include the prognostic factors closely related to the diagnosis of HCC (46). This indicates that the promotive role of m6A RNA methylation in the malignant progression of HCC might be played through the above signaling pathways. However, the specific molecular mechanisms, especially the interactions between m6A methylation regulator genes and the genes involved in the PPAR pathway as well as retinol metabolism, still require further study.

Current studies explore the role of m6A RNA methylation in tumor progression and prognosis. Accumulating studies have found that m6A RNA methylation regulatory factors can affect the prognosis of bladder cancer (47), head and neck squamous cell carcinoma (42), gastric cancer (40), colorectal adenocarcinoma (41), and other cancers. Here, we explored the correlation between m6A RNA methylation regulatory factors and the prognosis of HCC, and found that the expression levels of METTL3, YTHDF1 and YTHDF2 were related to the OS of HCC patients, while patients with high expression often have poor prognosis. Additionally, the expression levels of METTL3 and YTHDF1 also had a significant impact on DFS, suggesting that METTL3 and YTHDF1 can be used as independent prognostic factors in patients with HCC.

In conclusion, we systematically studied the expression, potential function and prognostic value of m6A RNA methylation regulatory factors in HCC, providing important evidence for further studying the role of RNA m6A methylation in HCC.

## Data Availability Statement

The datasets presented in this study can be found in online repositories. The names of the repository/repositories and accession number(s) can be found in the article/supplementary material.

## Author Contributions

ZZ, LZ, and SF contributed to the study design. LY conducted the literature search. FW, WC, JJ, and MC acquired the data. ZZ, LZ, and SF wrote the article. JS performed data analysis. LY drafted. FW, WC, and JJ revised the article and gave the final approval of the version to be submitted. All authors read and approved the final manuscript.

## Conflict of Interest

The authors declare that the research was conducted in the absence of any commercial or financial relationships that could be construed as a potential conflict of interest.
